# Draft genome sequence of soil isolate *Mycolicibacterium fortuitum* DVD-1301

**DOI:** 10.1128/MRA.00708-23

**Published:** 2023-11-09

**Authors:** Eugeny Y. Bragin, Dmitry V. Dovbnya, Tanya V. Ivashina, Marina V. Donova

**Affiliations:** 1 Skryabin Institute of Biochemistry and Physiology of Microorganisms, Russian Academy of Sciences, Federal Research Center “Pushchino Center for Biological Research of the Russian Academy of Sciences,” Pushchino, Moscow, Russia; California State University San Marcos, San Marcos, California, USA

**Keywords:** genomics, *Mycolicibacterium*, draft genome

## Abstract

Some strains of *Mycolicibacterium* possess high sterol-oxidizing activity and are used in the pharmaceutical industry for the production of steroid precursors. Herein, we report a draft genome sequence of the soil-dwelling *Mycolicibacterium fortuitum* DVD-1301 isolated in the floodplain of the river Oka. The genome contains a full set of steroid catabolic genes.

## ANNOUNCEMENT

Bacteria capable of degrading or modifying steroids are distributed in different environments (1). Of maximum interest are soil-dwelling Actinobacteria of the genus *Mycolicibacterium*, which usually possess the systems of active transport of lipophilic compounds and high metabolic plasticity, thus providing effective transformation or degradation of exogenous steroid substrates (2).

The isolate DVD-1301 was obtained from the soil sample taken at the Oka River (Russia) floodplain (54°50′36″ N, 37°36′52″E) in July 2021. The sample (1 g) was suspended in 10 mL of sterile saline, serially diluted up to 10^−6^, and incubated on R-2A agar (Sigma-Aldrich, USA) for 10 days at 30°C. The isolate was preliminarily identified as *Mycolicibacterium fortuitum* DVD-1301 based on the phenotype features and 16S rRNA sequence (3, 4). Genomic DNA was isolated as described earlier (5). Briefly, the cells were precipitated and sequentially treated with lysozyme (20 min, 37°C), SDS and proteinase K (1.0 h, 56°C), RNase A (30 min, 37°C) and then extracted with phenol-chloroform. Genome sequencing was performed by Illumina HiSeq 2000, as a result, 14,512,443 read pairs (length 2 × 100) were obtained. The Illumina library preparation was made by the KAPA DNA library preparation kit for Illumina and the KAPA dual-indexed adapter kit (Kapa Biosystems); the Illumina HiSeq SBS kit v3 was used for sequencing. Adapter and quality trimming was done by Trimmomatic 0.39 (6) with the settings ILLUMINACLIP:TruSeq3-PE:2:30:10:2, LEADING:3, TRAILING:3, and MINLEN:50. *De novo* genome assembly was made with the Ray 2.3.1 (7) program (-n 80 -k 27). In all cases where program parameters were not specified, default parameters were used.

The length of the sequenced genome is 6,726,749 nucleotides, included in 147 contigs (only contigs longer than 500 nucleotides were counted) with N50 equal to 278,938 and the G + C content is 72.66%. Annotation of the genome with NCBI PGAP (8) revealed 6,657 protein-coding genes, 64 RNA-coding genes; and 119 genes were annotated as pseudogenes. The NCBI database contains 44 genomes of various strains of *M. fortuitum*, the length of which varied from 6,126,360 (*M. fortuitum* E2981) to 7,279,489 (*M. fortuitum* ET2022-2333). The average nucleotide identity (ANI) between DVD-1301 and another strain of this species calculated with ANI Calculator (9) varies from 97.0 (*M. fortuitum* E3377) to 98.95 (*M. fortuitum* MTB7), on average 98.29. ANI between DVD-1301 and the type strain of *M. fortuitum* DSM 46621 was 98.69; the degree of DNA–DNA hybridization between these strains, calculated *in silico* with Genome-to-Genome Distance Calculator (http://ggdc.dsmz.de/home.php), was from 89 to 91.5%, depending on the formula used. This confirms that the strain DVD-1301 belongs to the *M. fortuitum* species.

**Fig 1:**
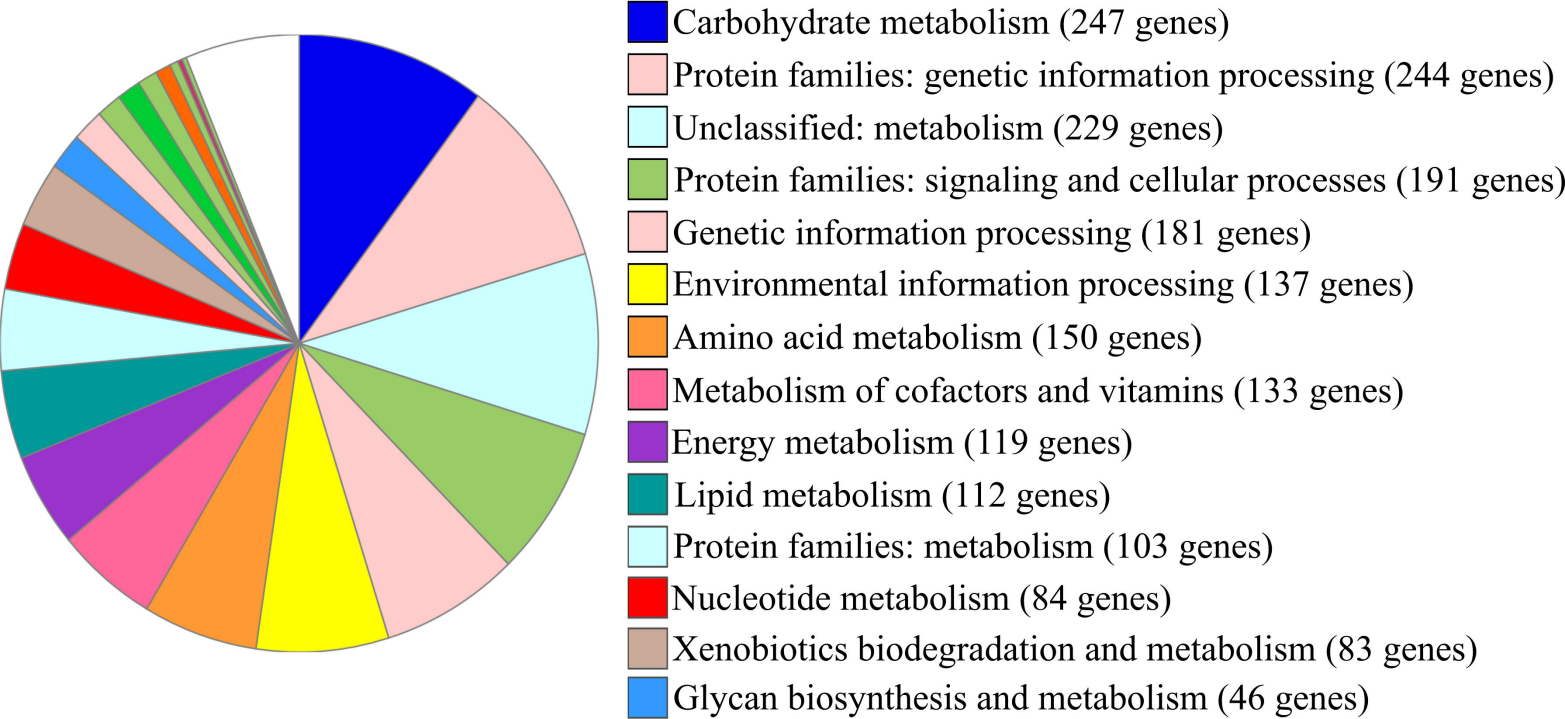
Functional groups of genes in *Mycolicibacterium fortuitum* DVD-1301 genome identified by BlastKOALA.

According to the search performed with BLAST+ 2.11.0 (10) within amino acid sequences from the GenBank database, the *M. fortuitum* DVD-1301 genome contains a complete set of genes of the core steroid catabolism. The number of genes related to various cellular functions determined with BlastKOALA (11) is presented in Fig. 1.

The data contribute to the knowledge of the diversity of steroid-transforming mycolicibacteria and may be of importance in creation of novel microbial catalysts for the production of valuable steroids.

## Data Availability

Raw genome reads have been uploaded to the SRA database (SRS17222831) and the genome sequences—to NCBI GenBank (JARUNL000000000.1). The BioProject is PRJNA952213, and the BioSample is SAMN34068755.
